# Supplementary figures and images for: Data-driven and interpretable machine-learning modeling to explore the fine-scale environmental determinants of malaria vectors biting rates in rural Burkina Faso
Source: Parasit Vectors. 2021 Jun 29;14:345. doi: 10.1186/s13071-021-04851-x (PMC8243492; doi:10.1186/s13071-021-04851-x)

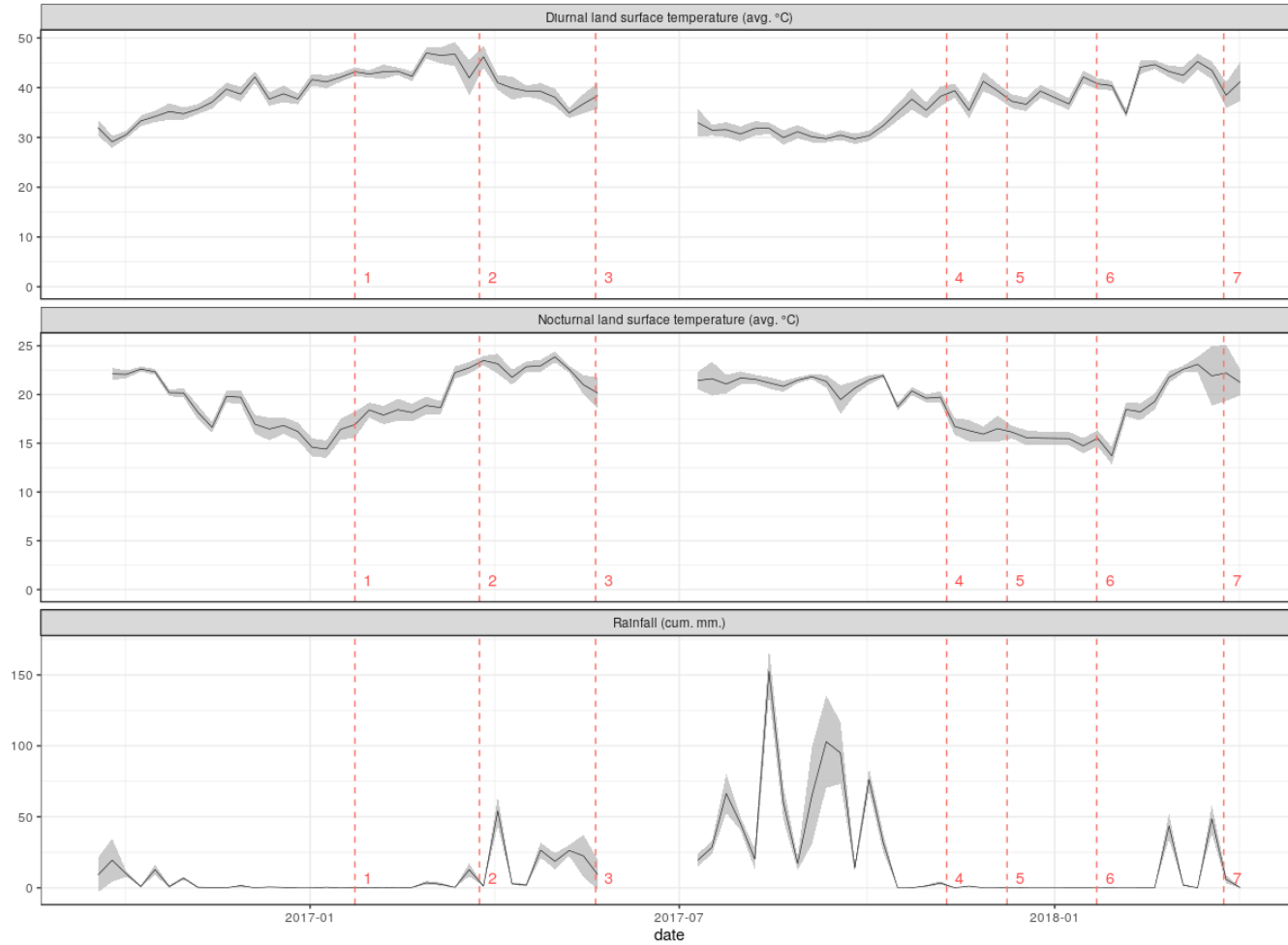

data sources : GPM (rainfall), MODIS (temperature)

Supplement: Supplementary file 1 — Additional file 1: Figure S1. Summary of the meteorological conditions around the sampling points. Average meteorological conditions in a 2 km radius buffer zone around the collection points (weekly aggregation). Vertical red lines indicate the dates of the entomological surveys. Ribbons indicate the mean ± one standard deviation considering all the sampling points for the week. [file 13071_2021_4851_MOESM1_ESM.pdf]

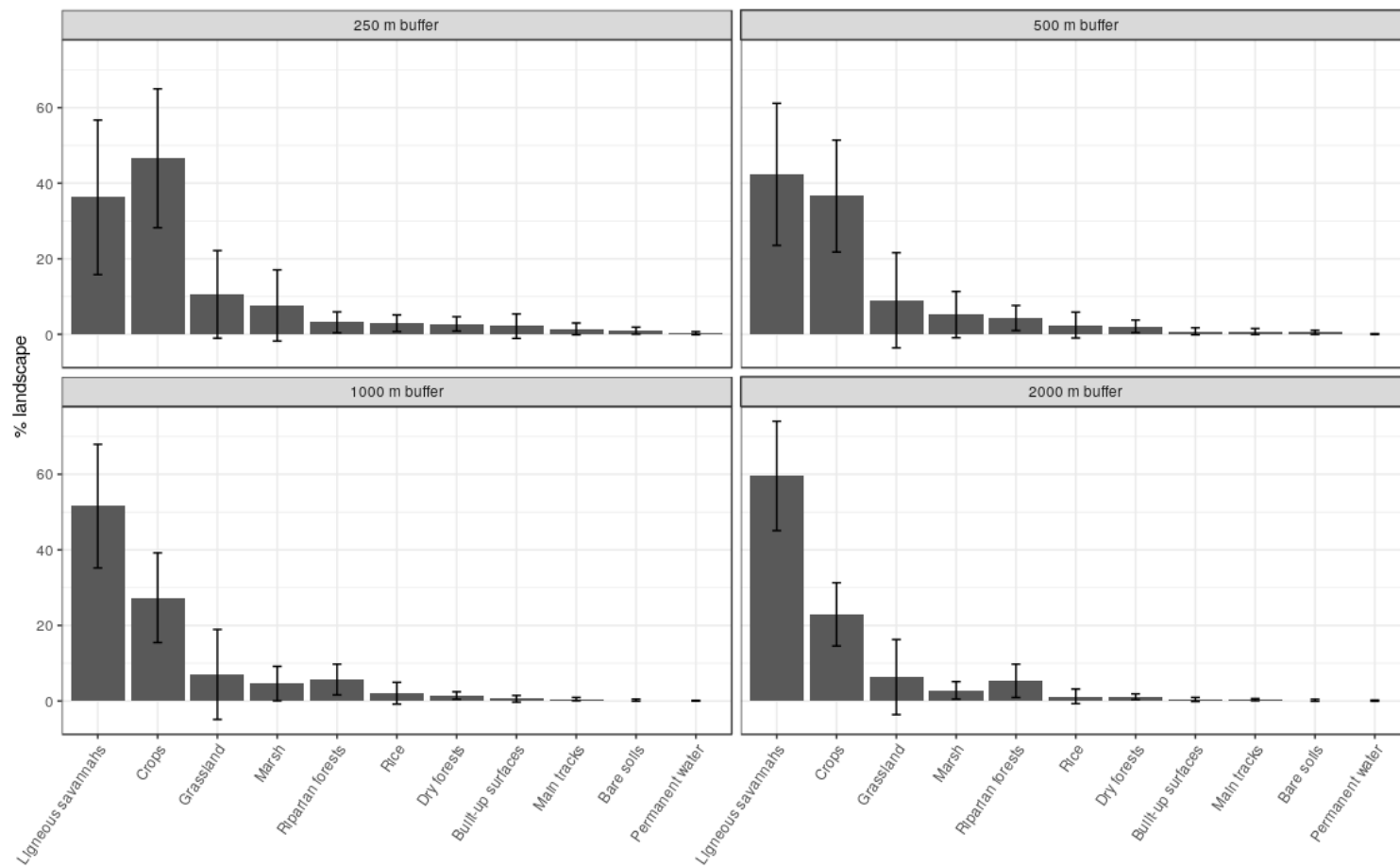

Data source : land cover map built from a supervised classification using satellite images

Supplement: Supplementary file 2 — Additional file 2: Figure S2. Summary of the landscape conditions around the sampling points. Average percentage of surface occupied by each land cover class in the various buffer zones (250 m, 500 m, 1 km, 2 km radii) around the collection points. Error bars indicate the mean ± one standard deviation considering all the sampling points. [file 13071_2021_4851_MOESM2_ESM.pdf]

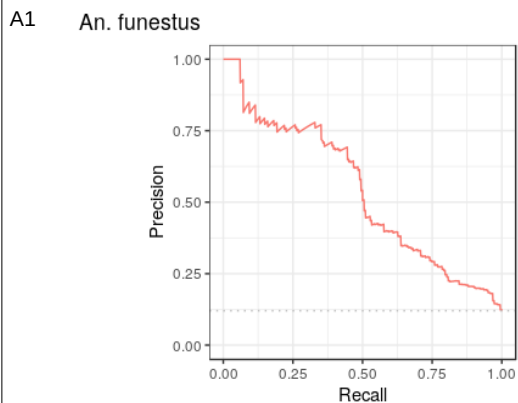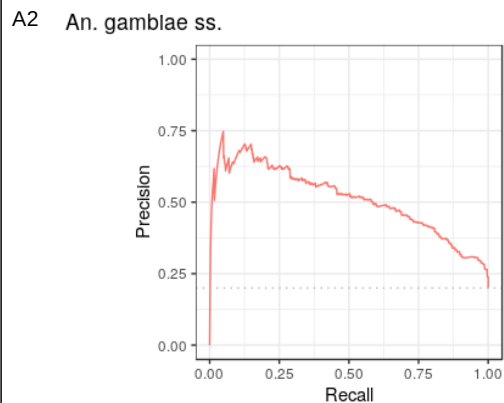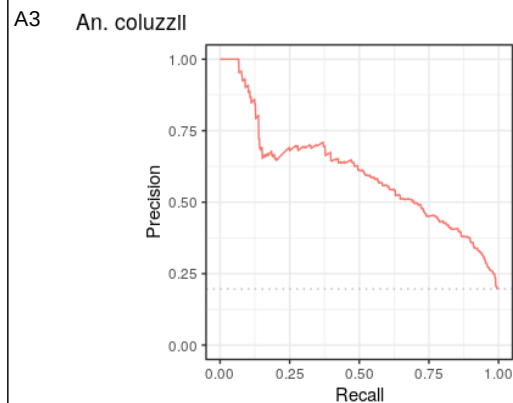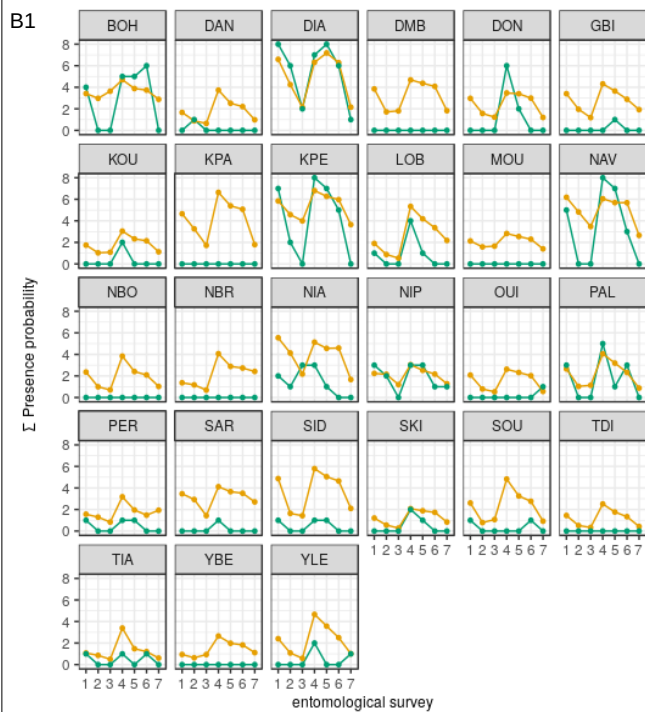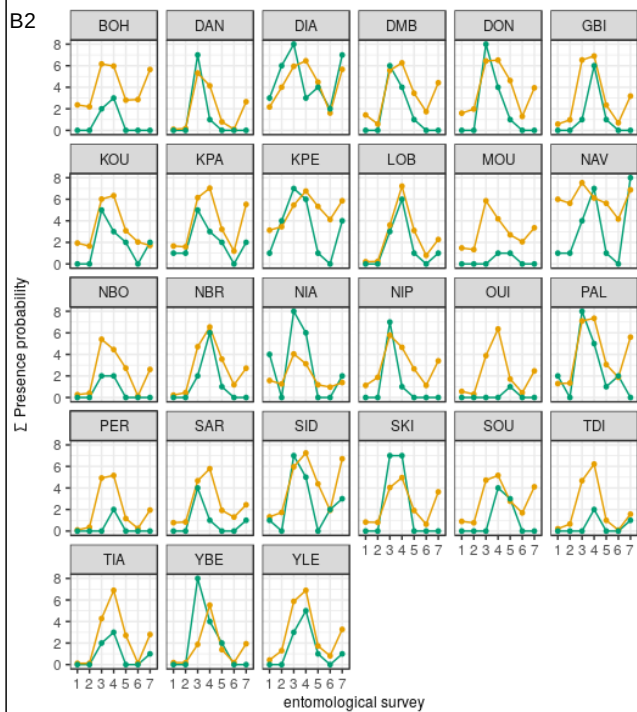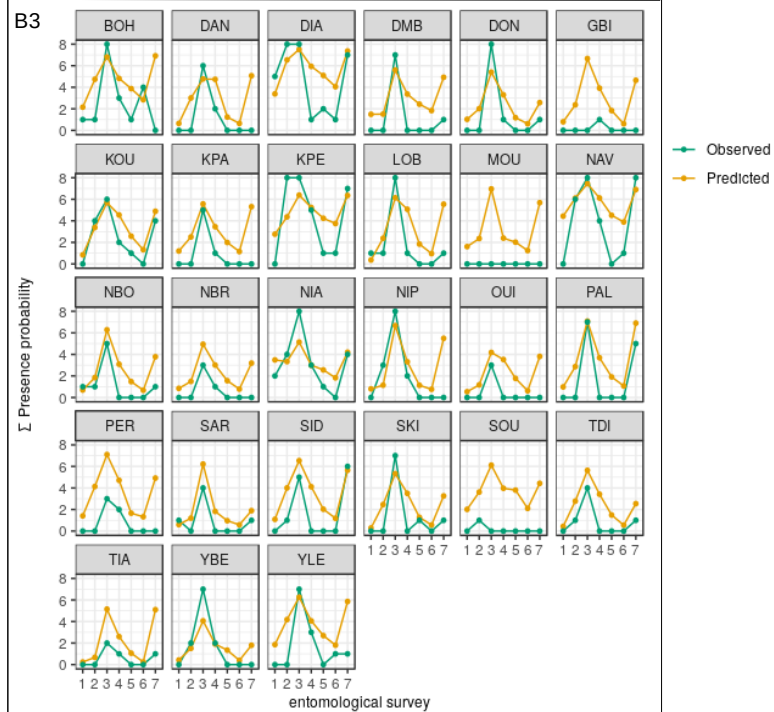

Supplement: Supplementary file 4 — Additional file 4: Figure S4. Model evaluation plots for the presence models. A1, A2, A3 are precision–recall curves for the presence models of respectively An. funestus, An. gambiae s.s. and An. coluzzii. Precision–recall curves show the precision and the recall of the models for different probability thresholds of the “presence” class. Precision is the proportion of presence identifications that was actually correct, while recall is the proportion of actual presence observations that were identified correctly. The horizontal dashed line represents the baseline (i.e. random or no-skill) classifier. A precision–recall curve above the horizontal line indicates a better-than-no-skill classifier. The higher the area between the precision–recall curve and the horizontal line, the better the classifier. Plots B1, B2, B3 are observed vs. predicted presence probabilities for each out-of-sample village. The y-axis represents the sum over the 8 sampling points/village/survey (4 points by village * 2 places (interior and exterior)). Overall, the plots A1, A2, A3 show that the models had good predictive accuracies (precision–recall curves are higher than the baseline curve, particularly for An. funestus and An. coluzzii). The plots B1, B2, B3 show that the models predicted well the spatiotemporal trends of presence/absence of bites (lines of predicted presence probabilities are generally close to lines of observed probabilities), although they usually slightly overestimated the probabilities of being bitten (predicted presence probability > observed presence probability). [file 13071_2021_4851_MOESM4_ESM.pdf]

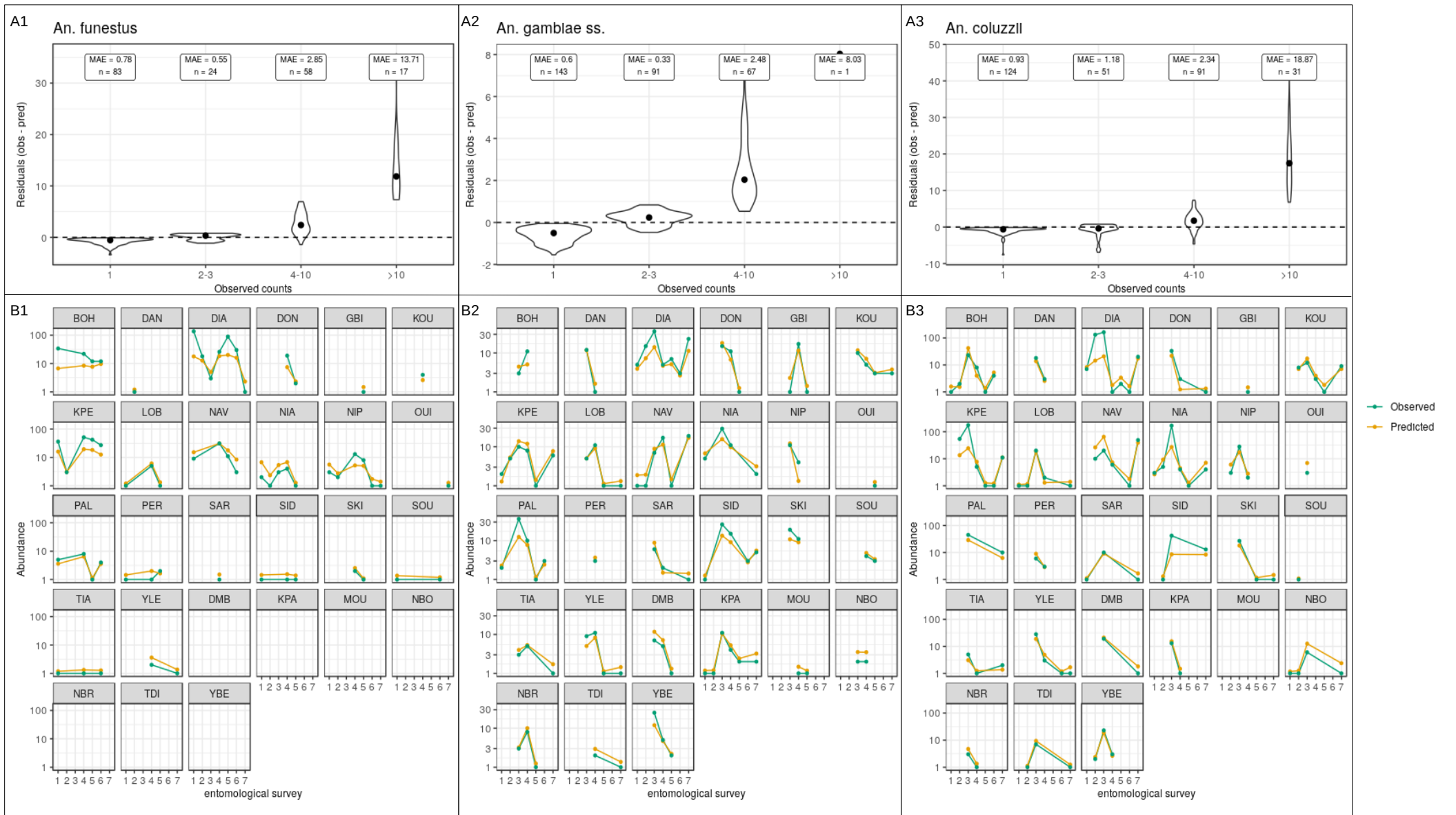

Supplement: Supplementary file 5 — Additional file 5: Figure S5. Model evaluation plots for the abundance models. A1, A2, A3 are violin plots of the distribution of the residuals for the abundance models of respectively An. funestus, An. gambiae s.s. and An. coluzzii, by observed counts of bites (4 classes: 1 bite, 2–3 bites, 4–10 bites, > 10 bites). Black dots indicate the median value. B1, B2, B3 are observed vs. predicted number of bites/village/entomological surveys. The y-axis represents the sum of bites over the 8 sampling points/village/survey (4 points by village * 2 places (interior and exterior)) on a logarithmic scale. The absence of a dot indicates that no vector was collected. MAE = mean absolute error; n = number of observations. Overall, the plots A1, A2, A3 show that the models predicted well small observed counts of bites (1 bite, 2–3 bites) (cf. small MAEs, small residuals), which represent the vast majority of observations (high n). Larger counts (4–10 bites, > 10 bites) tended to be underestimated by the models, especially for An. funestus and An. gambiae s.s. However, large counts (> 10 bites) represented few observations (small n). The plots B1, B2, B3 confirm these observations, and additionally show that general trends of biting rates over time were well predicted by the models (lines of predicted abundance are generally close to lines of observed abundance). [file 13071_2021_4851_MOESM5_ESM.pdf]
