# Supplementary material for: Data-driven and interpretable machine-learning modeling to explore the fine-scale environmental determinants of malaria vectors biting rates in rural Burkina Faso
Source: Parasit Vectors. 2021 Jun 29;14:345. doi: 10.1186/s13071-021-04851-x (PMC8243492; doi:10.1186/s13071-021-04851-x)
Supplement: Supplementary file 3 — Additional file 3: Figure S3. Pictures representative of the main land cover classes in the Diébougou area. Pictures were taken in November 2018. [file 13071_2021_4851_MOESM3_ESM.pdf]

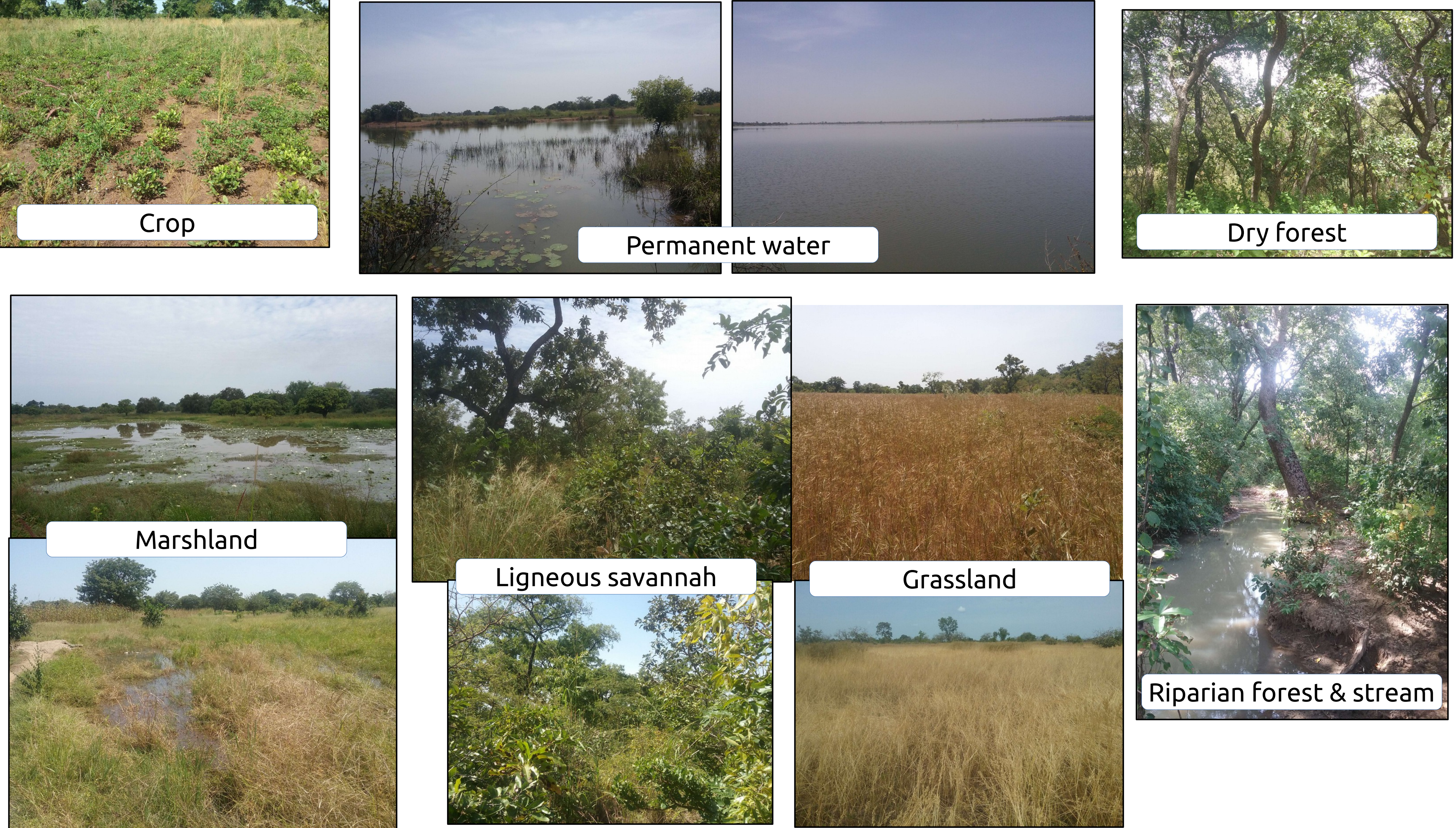
Crop
Permanent water
Dry forest
Marshland
Ligneous savannah
Grassland
Riparian forest & stream
