# Supplementary material for: Data-driven and interpretable machine-learning modeling to explore the fine-scale environmental determinants of malaria vectors biting rates in rural Burkina Faso
Source: Parasit Vectors. 2021 Jun 29;14:345. doi: 10.1186/s13071-021-04851-x (PMC8243492; doi:10.1186/s13071-021-04851-x)
Supplement: Supplementary file 6 — Additional file 6: Figure S6. Feature selection for the multivariate models. The figure shows the Spearman correlation coefficient between the explanatory variables and each response variable (presence and abundance of An. funestus, An. gambiae s.s. and An. coluzzii). Based on these results, variables were retained for the multivariate models according to the following criteria: we first excluded variables that were poorly correlated with the response variable (i.e. correlation coefficients less than 0.1 or p-values greater than 0.2 at all time associations or buffer radii considered), except for variables related to the presence of water—i.e. possible breeding sites—that were all retained whatever their correlation. Then, for each meteorological (resp. landscape) variable, we retained the time lag interval (resp. buffer radius) showing the higher absolute correlation coefficient value. We finally excluded collinear variables (i.e. Pearson correlation coefficient between the variables > 0.7) based on empirical knowledge. [file 13071_2021_4851_MOESM6_ESM.pdf]

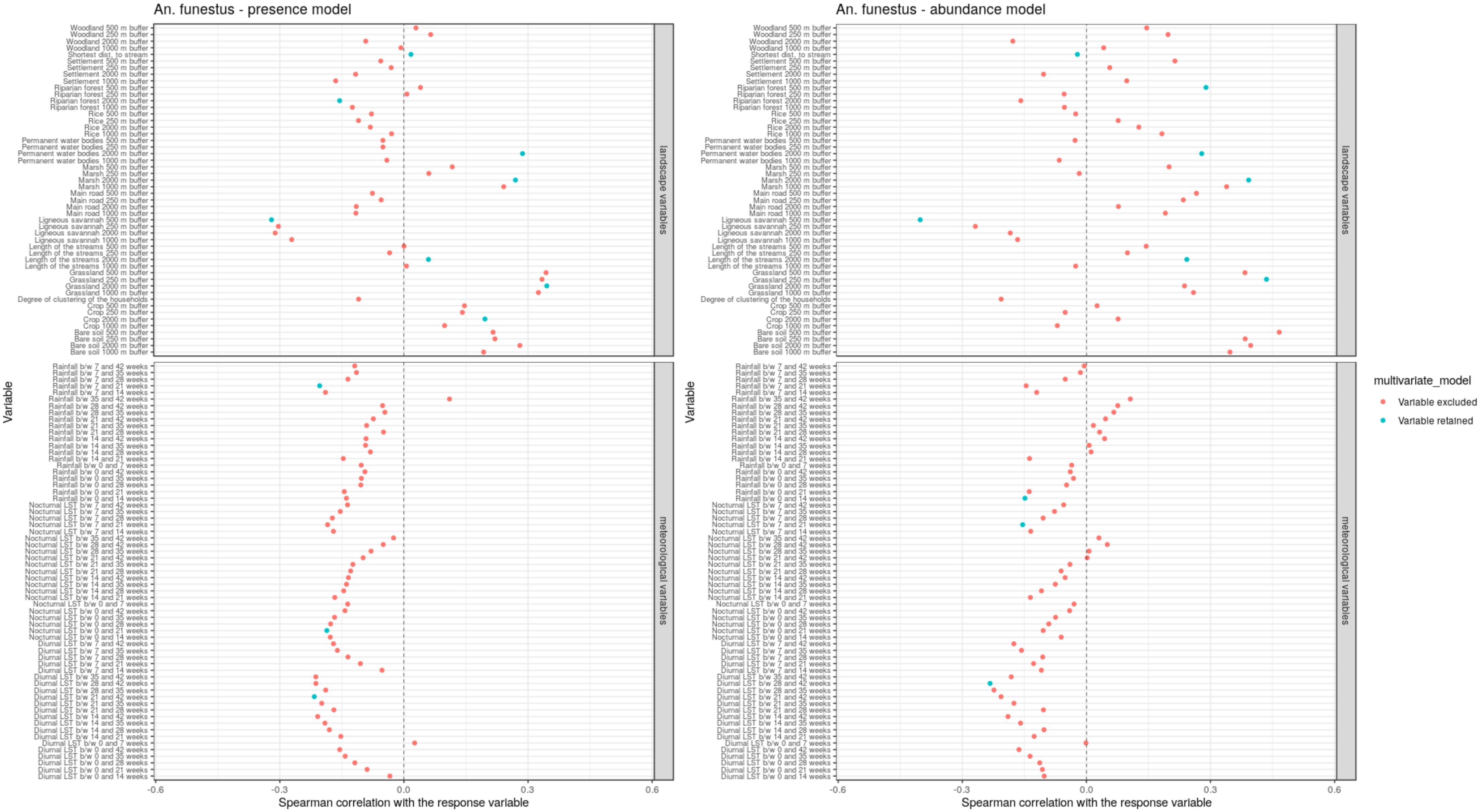
An. funestus - presence model
An. funestus - abundance model
Woodland 500 m buffer
Woodland 250 m buffer
Woodland 2000 m buffer
Woodland 1000 m buffer
Shortest dist. to stream
Settlement 500 m buffer
Settlement 250 m buffer
Settlement 2000 m buffer
Settlement 1000 m buffer
Riparian forest 500 m buffer
Riparian forest 250 m buffer
Riparian forest 2000 m buffer
Riparian forest 1000 m buffer
Rice 500 m buffer
Rice 250 m buffer
Rice 2000 m buffer
Rice 1000 m buffer
Permanent water bodies 500 m buffer
Permanent water bodies 250 m buffer
Permanent water bodies 2000 m buffer
Permanent water bodies 1000 m buffer
Marsh 500 m buffer
Marsh 250 m buffer
Marsh 2000 m buffer
Marsh 1000 m buffer
Main road 500 m buffer
Main road 250 m buffer
Main road 2000 m buffer
Main road 1000 m buffer
Ligneous savannah 500 m buffer
Ligneous savannah 250 m buffer
Ligneous savannah 2000 m buffer
Ligneous savannah 1000 m buffer
Length of the streams 500 m buffer
Length of the streams 250 m buffer
Length of the streams 2000 m buffer
Length of the streams 1000 m buffer
Grassland 500 m buffer
Grassland 250 m buffer
Grassland 2000 m buffer
Grassland 1000 m buffer
Degree of clustering of the households
Crop 500 m buffer
Crop 250 m buffer
Crop 2000 m buffer
Crop 1000 m buffer
Bare soil 500 m buffer
Bare soil 250 m buffer
Bare soil 2000 m buffer
Bare soil 1000 m buffer
Rainfall b/w 7 and 42 weeks
Rainfall b/w 7 and 35 weeks
Rainfall b/w 7 and 28 weeks
Rainfall b/w 7 and 21 weeks
Rainfall b/w 7 and 14 weeks
Rainfall b/w 35 and 42 weeks
Rainfall b/w 28 and 42 weeks
Rainfall b/w 28 and 35 weeks
Rainfall b/w 21 and 42 weeks
Rainfall b/w 21 and 35 weeks
Rainfall b/w 21 and 28 weeks
Rainfall b/w 14 and 42 weeks
Rainfall b/w 14 and 35 weeks
Rainfall b/w 14 and 28 weeks
Rainfall b/w 14 and 21 weeks
Rainfall b/w 0 and 7 weeks
Rainfall b/w 0 and 42 weeks
Rainfall b/w 0 and 35 weeks
Rainfall b/w 0 and 28 weeks
Rainfall b/w 0 and 21 weeks
Rainfall b/w 0 and 14 weeks
Nocturnal LST b/w 7 and 42 weeks
Nocturnal LST b/w 7 and 35 weeks
Nocturnal LST b/w 7 and 28 weeks
Nocturnal LST b/w 7 and 21 weeks
Nocturnal LST b/w 7 and 14 weeks
Nocturnal LST b/w 35 and 42 weeks
Nocturnal LST b/w 28 and 42 weeks
Nocturnal LST b/w 28 and 35 weeks
Nocturnal LST b/w 21 and 42 weeks
Nocturnal LST b/w 21 and 35 weeks
Nocturnal LST b/w 21 and 28 weeks
Nocturnal LST b/w 14 and 42 weeks
Nocturnal LST b/w 14 and 35 weeks
Nocturnal LST b/w 14 and 28 weeks
Nocturnal LST b/w 14 and 21 weeks
Nocturnal LST b/w 0 and 7 weeks
Nocturnal LST b/w 0 and 42 weeks
Nocturnal LST b/w 0 and 35 weeks
Nocturnal LST b/w 0 and 28 weeks
Nocturnal LST b/w 0 and 21 weeks
Nocturnal LST b/w 0 and 14 weeks
Diurnal LST b/w 7 and 42 weeks
Diurnal LST b/w 7 and 35 weeks
Diurnal LST b/w 7 and 28 weeks
Diurnal LST b/w 7 and 21 weeks
Diurnal LST b/w 7 and 14 weeks
Diurnal LST b/w 35 and 42 weeks
Diurnal LST b/w 28 and 42 weeks
Diurnal LST b/w 28 and 35 weeks
Diurnal LST b/w 21 and 42 weeks
Diurnal LST b/w 21 and 35 weeks
Diurnal LST b/w 21 and 28 weeks
Diurnal LST b/w 14 and 42 weeks
Diurnal LST b/w 14 and 35 weeks
Diurnal LST b/w 14 and 28 weeks
Diurnal LST b/w 14 and 21 weeks
Diurnal LST b/w 0 and 7 weeks
Diurnal LST b/w 0 and 42 weeks
Diurnal LST b/w 0 and 35 weeks
Diurnal LST b/w 0 and 28 weeks
Diurnal LST b/w 0 and 21 weeks
Diurnal LST b/w 0 and 14 weeks
landscape variables
meteorological variables
Variable
-0.6
-0.3
0.0
0.3
0.6
Spearman correlation with the response variable
multivariate_model
Variable excluded
Variable retained

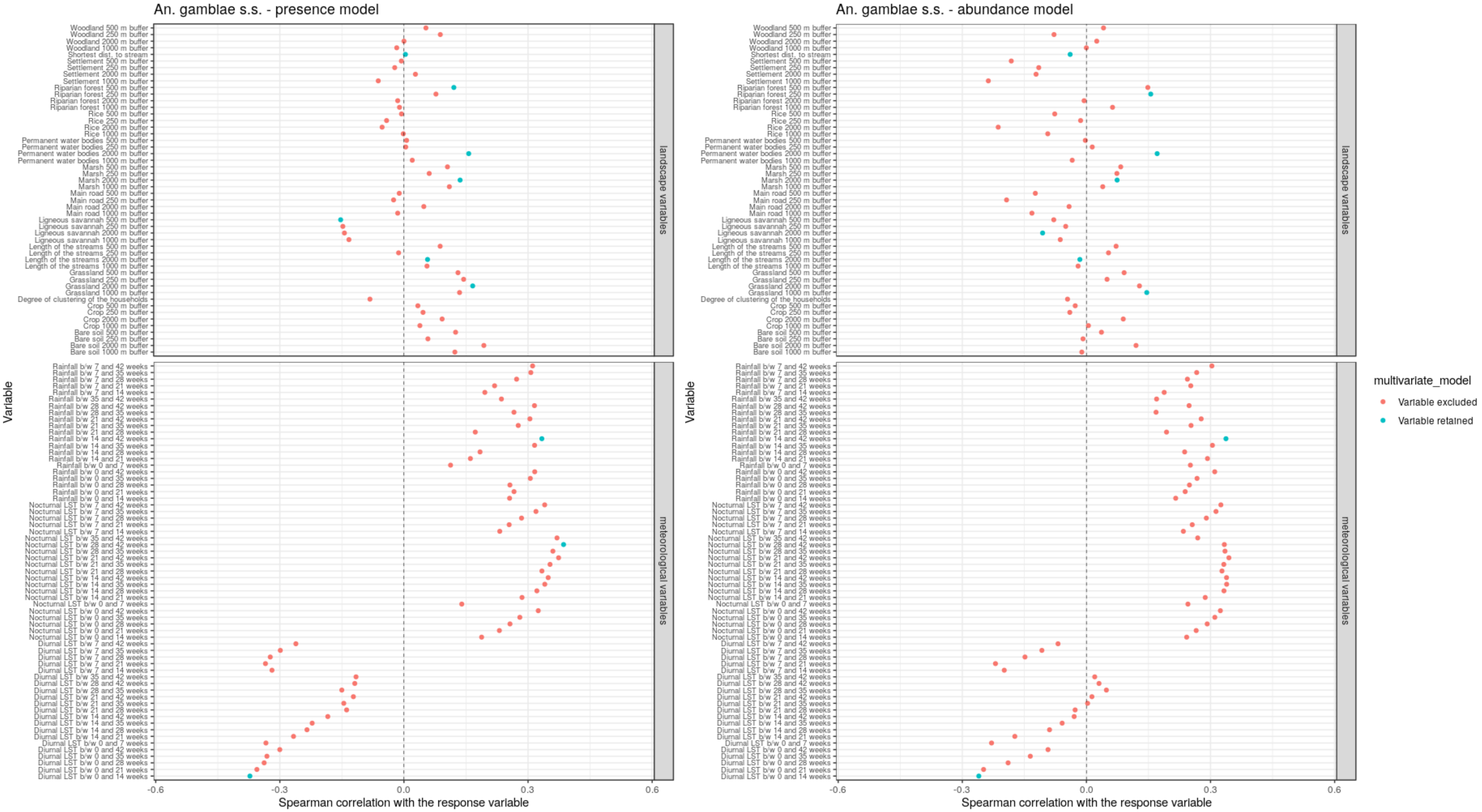
An. gambiae s.s. - presence model
An. gambiae s.s. - abundance model
Woodland 500 m buffer
Woodland 250 m buffer
Woodland 2000 m buffer
Woodland 1000 m buffer
Shortest dist. to stream
Settlement 500 m buffer
Settlement 250 m buffer
Settlement 2000 m buffer
Settlement 1000 m buffer
Riparian forest 500 m buffer
Riparian forest 250 m buffer
Riparian forest 2000 m buffer
Riparian forest 1000 m buffer
Rice 500 m buffer
Rice 250 m buffer
Rice 2000 m buffer
Rice 1000 m buffer
Permanent water bodies 500 m buffer
Permanent water bodies 250 m buffer
Permanent water bodies 2000 m buffer
Permanent water bodies 1000 m buffer
Marsh 500 m buffer
Marsh 250 m buffer
Marsh 2000 m buffer
Marsh 1000 m buffer
Main road 500 m buffer
Main road 250 m buffer
Main road 2000 m buffer
Main road 1000 m buffer
Ligneous savannah 500 m buffer
Ligneous savannah 250 m buffer
Ligneous savannah 2000 m buffer
Ligneous savannah 1000 m buffer
Length of the streams 500 m buffer
Length of the streams 250 m buffer
Length of the streams 2000 m buffer
Length of the streams 1000 m buffer
Grassland 500 m buffer
Grassland 250 m buffer
Grassland 2000 m buffer
Grassland 1000 m buffer
Degree of clustering of the households
Crop 500 m buffer
Crop 250 m buffer
Crop 2000 m buffer
Crop 1000 m buffer
Bare soil 500 m buffer
Bare soil 250 m buffer
Bare soil 2000 m buffer
Bare soil 1000 m buffer
Rainfall b/w 7 and 42 weeks
Rainfall b/w 7 and 35 weeks
Rainfall b/w 7 and 28 weeks
Rainfall b/w 7 and 21 weeks
Rainfall b/w 7 and 14 weeks
Rainfall b/w 35 and 42 weeks
Rainfall b/w 28 and 42 weeks
Rainfall b/w 28 and 35 weeks
Rainfall b/w 21 and 42 weeks
Rainfall b/w 21 and 35 weeks
Rainfall b/w 21 and 28 weeks
Rainfall b/w 14 and 42 weeks
Rainfall b/w 14 and 35 weeks
Rainfall b/w 14 and 28 weeks
Rainfall b/w 14 and 21 weeks
Rainfall b/w 0 and 7 weeks
Rainfall b/w 0 and 42 weeks
Rainfall b/w 0 and 35 weeks
Rainfall b/w 0 and 28 weeks
Rainfall b/w 0 and 21 weeks
Rainfall b/w 0 and 14 weeks
Nocturnal LST b/w 7 and 42 weeks
Nocturnal LST b/w 7 and 35 weeks
Nocturnal LST b/w 7 and 28 weeks
Nocturnal LST b/w 7 and 21 weeks
Nocturnal LST b/w 7 and 14 weeks
Nocturnal LST b/w 35 and 42 weeks
Nocturnal LST b/w 28 and 42 weeks
Nocturnal LST b/w 28 and 35 weeks
Nocturnal LST b/w 21 and 42 weeks
Nocturnal LST b/w 21 and 35 weeks
Nocturnal LST b/w 21 and 28 weeks
Nocturnal LST b/w 14 and 42 weeks
Nocturnal LST b/w 14 and 35 weeks
Nocturnal LST b/w 14 and 28 weeks
Nocturnal LST b/w 14 and 21 weeks
Nocturnal LST b/w 0 and 7 weeks
Nocturnal LST b/w 0 and 42 weeks
Nocturnal LST b/w 0 and 35 weeks
Nocturnal LST b/w 0 and 28 weeks
Nocturnal LST b/w 0 and 21 weeks
Nocturnal LST b/w 0 and 14 weeks
Diurnal LST b/w 7 and 42 weeks
Diurnal LST b/w 7 and 35 weeks
Diurnal LST b/w 7 and 28 weeks
Diurnal LST b/w 7 and 21 weeks
Diurnal LST b/w 7 and 14 weeks
Diurnal LST b/w 35 and 42 weeks
Diurnal LST b/w 28 and 42 weeks
Diurnal LST b/w 28 and 35 weeks
Diurnal LST b/w 21 and 42 weeks
Diurnal LST b/w 21 and 35 weeks
Diurnal LST b/w 21 and 28 weeks
Diurnal LST b/w 14 and 42 weeks
Diurnal LST b/w 14 and 35 weeks
Diurnal LST b/w 14 and 28 weeks
Diurnal LST b/w 14 and 21 weeks
Diurnal LST b/w 0 and 7 weeks
Diurnal LST b/w 0 and 42 weeks
Diurnal LST b/w 0 and 35 weeks
Diurnal LST b/w 0 and 28 weeks
Diurnal LST b/w 0 and 21 weeks
Diurnal LST b/w 0 and 14 weeks
landscape variables
meteorological variables
Variable
-0.6
-0.3
0.0
0.3
0.6
Spearman correlation with the response variable
multivariate_model
Variable excluded
Variable retained

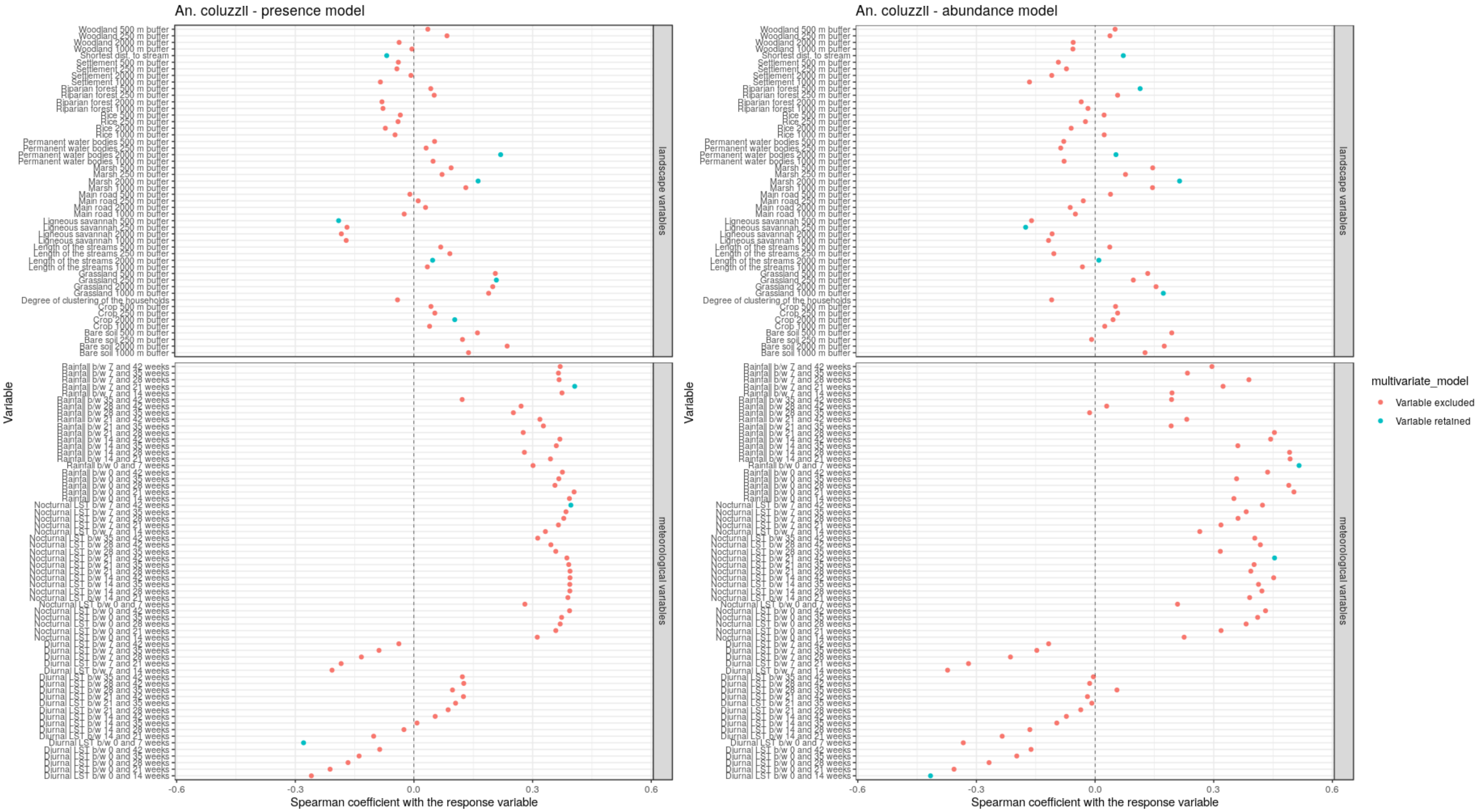

An. coluzzii - presence model
An. coluzzii - abundance model
Variable
Woodland 500 m buffer
Woodland 250 m buffer
Woodland 2000 m buffer
Woodland 1000 m buffer
Shortest dist. to stream
Settlement 500 m buffer
Settlement 250 m buffer
Settlement 2000 m buffer
Settlement 1000 m buffer
Riparian forest 500 m buffer
Riparian forest 250 m buffer
Riparian forest 2000 m buffer
Riparian forest 1000 m buffer
Rice 500 m buffer
Rice 250 m buffer
Rice 2000 m buffer
Rice 1000 m buffer
Permanent water bodies 500 m buffer
Permanent water bodies 250 m buffer
Permanent water bodies 2000 m buffer
Permanent water bodies 1000 m buffer
Marsh 500 m buffer
Marsh 250 m buffer
Marsh 2000 m buffer
Marsh 1000 m buffer
Main road 500 m buffer
Main road 250 m buffer
Main road 2000 m buffer
Main road 1000 m buffer
Ligneous savannah 500 m buffer
Ligneous savannah 250 m buffer
Ligneous savannah 2000 m buffer
Ligneous savannah 1000 m buffer
Length of the streams 500 m buffer
Length of the streams 250 m buffer
Length of the streams 2000 m buffer
Length of the streams 1000 m buffer
Grassland 500 m buffer
Grassland 250 m buffer
Grassland 2000 m buffer
Grassland 1000 m buffer
Degree of clustering of the households
Crop 500 m buffer
Crop 250 m buffer
Crop 2000 m buffer
Crop 1000 m buffer
Bare soil 500 m buffer
Bare soil 250 m buffer
Bare soil 2000 m buffer
Bare soil 1000 m buffer
landscape variables
Rainfall b/w 7 and 42 weeks
Rainfall b/w 7 and 35 weeks
Rainfall b/w 7 and 28 weeks
Rainfall b/w 7 and 21 weeks
Rainfall b/w 7 and 14 weeks
Rainfall b/w 35 and 42 weeks
Rainfall b/w 28 and 42 weeks
Rainfall b/w 28 and 35 weeks
Rainfall b/w 21 and 42 weeks
Rainfall b/w 21 and 35 weeks
Rainfall b/w 21 and 28 weeks
Rainfall b/w 14 and 42 weeks
Rainfall b/w 14 and 35 weeks
Rainfall b/w 14 and 28 weeks
Rainfall b/w 14 and 21 weeks
Rainfall b/w 0 and 7 weeks
Rainfall b/w 0 and 42 weeks
Rainfall b/w 0 and 35 weeks
Rainfall b/w 0 and 28 weeks
Rainfall b/w 0 and 21 weeks
Rainfall b/w 0 and 14 weeks
Nocturnal LST b/w 7 and 42 weeks
Nocturnal LST b/w 7 and 35 weeks
Nocturnal LST b/w 7 and 28 weeks
Nocturnal LST b/w 7 and 21 weeks
Nocturnal LST b/w 7 and 14 weeks
Nocturnal LST b/w 35 and 42 weeks
Nocturnal LST b/w 28 and 42 weeks
Nocturnal LST b/w 28 and 35 weeks
Nocturnal LST b/w 21 and 42 weeks
Nocturnal LST b/w 21 and 35 weeks
Nocturnal LST b/w 21 and 28 weeks
Nocturnal LST b/w 14 and 42 weeks
Nocturnal LST b/w 14 and 35 weeks
Nocturnal LST b/w 14 and 28 weeks
Nocturnal LST b/w 14 and 21 weeks
Nocturnal LST b/w 0 and 7 weeks
Nocturnal LST b/w 0 and 42 weeks
Nocturnal LST b/w 0 and 35 weeks
Nocturnal LST b/w 0 and 28 weeks
Nocturnal LST b/w 0 and 21 weeks
Nocturnal LST b/w 0 and 14 weeks
Diurnal LST b/w 7 and 42 weeks
Diurnal LST b/w 7 and 35 weeks
Diurnal LST b/w 7 and 28 weeks
Diurnal LST b/w 7 and 21 weeks
Diurnal LST b/w 7 and 14 weeks
Diurnal LST b/w 35 and 42 weeks
Diurnal LST b/w 28 and 42 weeks
Diurnal LST b/w 28 and 35 weeks
Diurnal LST b/w 21 and 42 weeks
Diurnal LST b/w 21 and 35 weeks
Diurnal LST b/w 21 and 28 weeks
Diurnal LST b/w 14 and 42 weeks
Diurnal LST b/w 14 and 35 weeks
Diurnal LST b/w 14 and 28 weeks
Diurnal LST b/w 14 and 21 weeks
Diurnal LST b/w 0 and 7 weeks
Diurnal LST b/w 0 and 42 weeks
Diurnal LST b/w 0 and 35 weeks
Diurnal LST b/w 0 and 28 weeks
Diurnal LST b/w 0 and 21 weeks
Diurnal LST b/w 0 and 14 weeks
meteorological variables
-0.6
-0.3
0.0
0.3
0.6
Spearman coefficient with the response variable
multivariate_model
Variable excluded
Variable retained
